# Gender Difference in General Self-Efficacy among Young-Old Elderly Aged 60–74 in Rural Shandong China: A Cross-Sectional Survey

**DOI:** 10.3390/ijerph16245070

**Published:** 2019-12-12

**Authors:** Yali Wang, Lingzhong Xu, Wenzhe Qin, Jiao Zhang, Yu Xia, Xiang Jing, Lu Lu, An’an Jiao, Yaozu Li

**Affiliations:** 1 School of Public Health, Shandong University, Jinan 250012, China; wyl_0719@163.com (Y.W.); qinwenzhe09@163.com (W.Q.); 201614324@mail.sdu.edu.cn (J.Z.); xy978742748@foxmail.com (Y.X.); 13209897715@163.com (X.J.); lulu20170320@163.com (L.L.); jiaoanan0328@163.com (A.J.); liyaozu1211@163.com (Y.L.); 2 NHC Key Laboratory of Health Economics and Policy Research (Shandong University), Jinan 250012, China; 3 Shandong University Center for Health Economics Experiment and Public Policy Research, Jinan 250012, China

**Keywords:** general self-efficacy, gender difference, young-old, China

## Abstract

Objective: This study aims to explore the determinants of general self-efficacy (GSE) among young-old elderly, with focus on examining the gender difference of general self-efficacy. Methods: Data were collected from the 2017 Survey of the Shandong Elderly Family Health Service, which was conducted by Shandong University. T-test was used to examine the gender difference in GSE. Univariate models and adjusted multiple linear regression model were used to explore the determinants of GSE by gender. Results: The females’ GSE score was lower than that of male participants (26.1 ± 8.1 vs. 28.7 ± 7.7), and there was a significant gender difference (t = 10.877, *p* < 0.001). Multiple linear regression model showed that some factors are common significant determinants of GSE such as age, education level, activity of daily living (ADL), self-rated health, mental health, personality, and whether participants have intimate friends and interpersonal relationships. Hypertension and frequent communication with children were specific determinants of GSE among male young-old. Personal income was a specific determinant of female participants. Conclusion: Some influencing factors of GSE in both genders are identical, the others are different. More attention should be paid for the poor young-old females, young-old males with hypertension, and disabled young-old people.

## 1. Introduction

It is well established that the world population continues to grow older rapidly. A World Health Organization (WHO) report on aging and health pointed out that the proportion of the world’s population over 60 years of age will nearly double, from 12% to 22%, between 2015 and 2050 [1]. As the world’s most populated country, China also has the largest elderly population size. In China, the percentage of people aged 65 years and above was 11.9% in 2018, which is predicted to reach 26.8% in 2050 [2]. This will inevitably give rise to a host of health and social challenges. As a result, the prevalence and incidence of age-associated diseases encompassing cancer, chronic non-communicable diseases, and mental health disorders are increasing [3]. In China, there are more than 100 million adults with diabetes, and over 177 million adults with hypertension [4]. A cross-sectional survey also disclosed that over 39% of elders had self-reported depressive symptoms [5]. Maintaining physical and psychological health in old age becomes a public concern. Maintaining physical and mental health have crucial roles in healthy aging. General self-efficacy seems to be an essential factor in improving elderly mental and physical health.

General self-efficacy (GSE) is based on Bandura’s theory of self-efficacy, reflecting the degree of overall self-confidence that individuals when confronted and dealing with difficult situations [6]. According to research, self-efficacy influences the way individuals feel, think, self-motivate, and behave; high GSE prompts people to think positively, with a high sense of self-efficacy, and an individual responds more assertively in interaction with others [7,8]. In studies of elderly self-care, it has been identified as a significant predictor of successful self-management [9]. Empirical researches also confirm that it can highly increase the elderlys’ confidence to perform activities and overcome unique challenges, and a low sense of GSE is associated with depression, anxiety, and helplessness [10,11]. Meanwhile, GSE can improve the health behavior and health management of the elderly and has a significant effect on life quality of elderly [11,12,13,14,15].

According to related research, ‘the elderly’ is not a homogeneous group, and can be divided into 3 subgroups: Young-old, middle-old, and oldest-old [16,17,18,19]. There are a variety of age classification standards. We focused on the elderly aged 60–74 as young-old in combination with domestic and foreign studies [20,21,22]. The middle-old and oldest-old tend to focus on having peace of mind and the absence of anger, in spite of them being older and having more health-related problems, they rate their health more positively than the young-old, and they are also less affected by negative emotions than the young-old [23,24,25]. The young-old take up a considerable proportion of the elderly. They have just entered old age. With increasing age, physical functions decline, so their health condition is not as good as before, and they become more vulnerable to the adverse effects of negative interactions [26]. What is more, due to the one-child policy of China, most of the young-old have only one child [27]. At this stage, children leave home for an independent lives and the young-old become ‘empty nesters’ [28]. The lack of emotional comfort leads to low level of social support, so the young-old are prone to negative feelings such as inferiority and self-pity [29]. Therefore, facing the loss in physical, social, and other resources, the young-old may have more stress, which will lead to the reduction of GSE. One study also indicated GSE varies according to age, which increases throughout childhood and early adulthood, reaches its peak in middle age, and decreases after 60 years of age [30]. Therefore, it is worth paying attention to the GSE of the young-old.

Our study focused on the GSE of young-old people in Shandong Province. Shandong has the second largest population with the size of one hundred million in China, and the percentage of its elderly population (65+) has reached 14.0% by 2017 [31], compared with 19.7% in the United States (U.S.) and 27.7% in Japan [32,33]. Improving the GSE of the elderly in Shandong Province will have a positive effect on its healthy aging. The existing researches on GSE mainly focus on its effect and significance. Though available data shows there is gender difference on GSE among the young-old, no in-depth investigation followed [14,34]. The gender difference of GSE in the young-old is still unclear. To fill the research gap, our study aims to explore the gender difference of GSE among young-old and its determinants.

## 2. Materials and Methods 

### 2.1. Data and Sampling

Data were collected from the 2017 Survey of the Shandong Elderly Family Health Service, which was conducted by Shandong University [35,36]. The survey samples were extracted by multi-stage random sampling method. In the first stage, 6 counties were selected from 137 counties as the primary sampling units (PSUs) throughout the eastern, central, and western areas of Shandong Province (which were divided into 3 districts and 3 counties that represented urban and rural areas separately). From each PSU, 18 villages in the rural area and 18 communities in the suburban and urban area were selected as the secondary sampling units (SSUs). In the third stage, based on the roster of the residents by age and the total elderly population of each selected site provided by the local residential committee, an average of 66 individuals were stratified and randomly selected from each SSU making up the total sample. The eligible participants for this survey were those aged 60 or older with local household registrations at the time of the interview. Initially, 7088 elderly individuals were selected and interviewed. Of these, 18 did not complete the survey. In total, 7070 individuals were included in the final sample, including 5486 elderly aged 60–74, and we focused on respondents aged 60–74 in the rural area with a sample of 4293.

We collected the primary data in August 2017. All participants accepted about 30 minutes face-to-face interviews by trained postgraduate students from the school of public health, Shandong University. The study protocol was approved by the Ethical Committee of Shandong University School of Public Health. The students explained the purpose of the survey before the interview begun. Informed consent for the collection and use of information was obtained from all participants. To ensure quality, completed questionnaires were checked by quality supervisors.

### 2.2. Dependent Variable

General self-efficacy was measured by GSE scale [37]. Foreign scholars have compiled self-efficacy scales for different groups of people, we used the general self-efficacy scale as a measuring tool because our research object was the general population. The scale includes 10 items: Each item is rated on a four-point scale with numerical values assigned to the responses (not at all true, hardly true, moderately true, and exactly true), yielding a total score between 10 and 40. The higher the score, the higher the general self-efficacy. The Cronbach s α was 0.96 in this sample.

### 2.3. Independent Variable

#### 2.3.1. Socio-Demographic Characteristics

The socio-demographic characteristics included age, gender (male, female), marital status (married, others (latter one includes unmarried, divorced, and widowed)), education level (illiteracy, primary school, junior school and senior school or above), work status (employed, unemployed), personal income level (Q1, Q2, Q3, and Q4). Quartile 1 (Q1) is the poorest and Quartile 4 (Q4) is the richest. 

#### 2.3.2. Physical and Psychological Health Characteristics

The physical and psychological health included activity of daily living (ADL), self-rated health (good, normal, or bad), hypertension, diabetes, coronary, psychological distress (Kessler 10), personality (extrovert, introvert or both), living arrangements (alone or with children), frequent communication with children (yes vs. no), had intimate friends (yes vs. no), and satisfied with personal relationship (yes, normal or no). ADL was measured using the ADL subscales and instrumental activity of daily living (IADL) subscales. Scores for performing activities range from 1 to 4 (1 point for each activity performed without help and 4 points for each activity that the individuals unable to perform). The maximum score is 56 (higher scores indicate greater dependence). The total score of ADL can be divided into 3 levels: Level I refers to normal function (score < 14), level II refers to moderate dysfunction (score 14–22), level III refers to bad dysfunction (score > 22). Edwards confirmed the ADL scales in a Brazilian study, showing a Cronbach s α of 0.96–0.99 [38]. The Cronbach s α for the ADL was 0.93 in this sample.

Kessler 10 (K10) [39] was used to measure psychological distress. An individual’s sense of psychological distress is evaluated against ten items on a self-report questionnaire. Each item is rated on a five-point scale with numerical values assigned to the responses (none of the time, a little time, some of the time, most of the time, and all the time). The ratings are summed to yield a total score, with higher scores indicating higher levels of psychological distress. The scale’s internal consistency, as assessed by Cronbach s α, was 0.87 [40]. The Cronbach s α for the K10 was 0.92 in this sample.

### 2.4. Statistical Analysis

Data analyses were performed using SPSS24.0. Chi-square tests was used to testing relationships between categorical variables, t-test was used to compare the difference in continuous variables. Univariate models and adjusted multiple linear regression model were used to explore the determinants of GSE among elderly by gender. Model 1: univariate model. Model 2: multiple linear regression model, adjusted for demographic characteristics (age, marital status), socioeconomic status (education level and personal income), and physical and psychological health characteristics. All reported CIs were calculated at the 95% level. A *p* value of less than 0.05 was considered statistically significant for the test.

## 3. Results

### 3.1. Sample Description

Among the 4293 participants, 1800 (41.9%) were males, 2493 (58.1%) were females. Generally speaking, the majority of the participants were married (86.0%), more than one-third (42.2%) participants were primary school graduates, and nearly half were currently employed (46.4%). Half of the elderly had great self-rated health status (52.7%). The variables with significant difference between males and females were age, marital status, education level, work status, income, ADL, self-rated health, hypertension, diabetes, coronary, psychological distress, living arrangements, frequent communication with children, and had intimate friends (Table 1). The elderlys’ GSE score was 27.2 ± 8.1, with 28.7 ± 7.7 for males and 26.1 ± 8.1 for females. There was a significant gender difference in GSE (t = 10.877, *p* < 0.001).

### 3.2. Determinants of GSE among Male Young-Old Elderly

Table 2 shows the determinants of GSE among male young-old. The results indicated that the older the age, the lower the GSE; the higher the education level, the higher their GSE score; the GSE of males who had moderate or bad dysfunction was lower; the poorer the self-rated health, the lower the GSE score; the males with hypertension had a lower GSE than other males; those who had higher psychological distress had a lower GSE score; the GSE of extroverted males elderly was higher than that of introvert males elderly; the GSE score of males who always communicated with children were higher; the GSE of males who had intimate friends were higher; and the GSE of males who are dissatisfied with interpersonal relationship or had a moderate feeling was lower.

### 3.3. Determinants of GSE among Female Young-Old Elderly

Table 3 shows the female young-old who was older had a lower GSE score; as the level of education increases, the GSE of female elderly also increases; personal income was significantly associated with GSE; the GSE of females who had moderate or bad dysfunction was lower; the poorer the self-rated health of the female, the lower the GSE; the higher the psychological distress, the lower the GSE score; the extrovert females had higher GSE score than introvert females; the GSE of females who had intimate friends was higher than those who did not have them; and the GSE score of females who had a moderate feeling about interpersonal relationship was lower than those who were satisfied with interpersonal relationship.

## 4. Discussion

Previous studies had shown that GSE can promote elderly people to develop healthy living behaviors and help delay the development of diseases and complications [41], it plays a key role in the individual behavior change, and can improve the self-confidence and sense of achievement of the elderly. Therefore, enhancing the GSE of the elderly is of great significance for improving the quality of life of the elderly [12,13,42]. Shandong province is the birthplace of Confucian and Mencius, and Shandong rural areas are deeply influenced by Chinese traditional culture. For thousands of years, the concept of “men are more able” was deep-rooted [43]. Chinese males are supposed to learn how to be independent and ambitious because they are expected to play a dominant role in Chinese society. Females are taught to be dependent and to submit to males’ decisions. The traditional thinking is of importance in understanding gender difference [44], traditional gender roles may contribute to individual s GSE. It’s worthy to explore gender difference in GSE.

Our study found that males had significantly higher GSE than females. It is similar with the study of Wang Su-juan [45] and Panadero [34]. The first reason is that females have certain disadvantages in terms of role division and social resources, gendered family division, and social division of labor make females unable to obtain equal family discourse and socioeconomic status compared with males [46]. To a certain extent, this did harm to the right of female to obtain healthy resources in the family and society. This disadvantage will accumulate in the old age with the increase of age. Second, as a research shown that, males sometimes overestimate their abilities and performance when self-recognizing, while females were just reverse [47]. Therefore, males may have a relatively high level of confidence in facing challenges and therefore exhibit a higher level of GSE. Third, Shandong Province is the birthplace of Confucian and Mencius culture, the traditional thinking of “men are more able” was deeply rooted. Thus, males may feel obliged to present themselves in a positive light under the influence of social desirability. Individuals with high social desirability may deceive themselves and/or others in self-reports of behavior in order to make them look better than is actually the case [48]. Then they may be more inclined to have a high sense of GSE.

Our study found that among the female elderly, personal income was significantly positively associated with GSE, which was consistent with the finding of LONG Dandan’s study [49]. Elderly people with a high income have stronger self-confidence and were more likely to achieve goals and solve problems efficiently, but this relationship between income and GSE was not significant among male elderly. Maybe the reason is this is the income of females is often lower when compared to the males, especially in rural areas. Females have lower socioeconomic status than males [50,51], which was consistent with studies of Wang, Y. and Asadullah, M.N. [52,53]. As a result, the female is more sensitive when the income changes, so the impact of incomes on female GSE is more pronounced.

Among male elderly, hypertension was significantly associated with the GSE. Self-efficacy has been associated with better chronic disease self-care among individuals management [54]. Studies showed that the overall level of self-management in males with hypertension is lower than that in females, and they are inferior to female in the management of diet control and treatment compliance [55,56,57]. This gave females a belief in controlling disease, whereas males do not seem to have a strong belief in it. In consequence the male elderly with hypertension may have a low GSE. Further research is needed to explore.

Communication frequency with sons and daughters also significantly affects the GSE of male group, though it is not significant within the female group. This outcome is different from previous studies. Traditional belief suggests that female’s emotion is more abundant, their relationships with their children are more intimate, while the male are not good at expressing emotions. The emotional support has less impact on the welfare of male elderly than on female elderly [58,59]. However, most of the young-old in this study are parents of only one child which is different from previous studies. They are adapting to life after their children leave home. They need more care and communication with their children due to separation anxiety. It may be precisely because males are not good at expressing emotions that their relationships with their children are not intimate compared with females. Therefore, when male elderly have intimate parent–child relationships, they can show stronger self-confidence in life and may have higher sense of GSE, which in accordance with SONG Lu’s study [60].

In our study, those young-old with high level of education, normal daily activities, and good self-rated health status had higher level of GSE, which is consistent with most studies [61,62]. The reason may be that with the increase of age, the physiological and social resources of the elderly are lost, and their daily activities are limited. The elderly had no confidence in the rehabilitation and treatment of disease, and thus their GSE is reduced [63]. Elderly people with higher education have a higher literacy of things and a stronger ability to accept their circumstances. They believe that they have the ability to solve most problems, so they usually have higher sense of GSE [64].

Our study also found that the variables including being satisfied with interpersonal relationships, extroverted personality, having intimate friends, and lower psychological distress had a positive effect on GSE, which is consistent with the results of other studies [65,66]. Satisfaction with relationships and having intimate friends can reflect the level of social support, and social support has been shown to play a key role in the coping process enabling individuals to alter the way they view and experience their lives by engaging in a process of cognitive restructuring [67,68], thus providing another source of increased self-efficacy [69]. An individual with an extroverted personality and lower psychological distress tends to have fewer negative emotions, and GSE has shown inversely correlated with depression, anxiety, and lower mental health status [70]. In consequence, these people may have a higher GSE.

This study also had some limitations. First, the nature of the cross-sectional study led to a causal relationship that could not be confirmed. Second, there may be more confounding factors than those available for consideration in this study. Third, the inherent characteristics of the elderly such as hearing impairment and memory loss may lead to information bias.

## 5. Conclusions

In conclusion, our study found that the GSE of the male young-old was higher than that of the females. The results of this study highlight the gender difference in GSE among rural young-old and the necessary to better understand the determinants of GSE. The common determinants for GSE of rural young-old were age, education level, activity of daily living (ADL), self-rated health, psychological distress, personality, and whether they have intimate friends and interpersonal relationships. We also found that personal income was significantly correlated with GSE in females but not in males. Hypertension and frequent communication with children were specific determinants of GSE among males. Health policies should focus on the psychological health of the young-old and enabling them to get a higher GSE, and gender difference should also be considered. More attention can be paid to vulnerable groups such as males with hypertension and poor females through the work of village committees. Psychosocial care is also essential for the elderly. At the same time, children should pay attention to the emotional needs of the elderly while giving their support obligations, and give parents, especially fathers, emotional companionship and support.

## Figures and Tables

**Table 1:** Characteristics of young-old in rural Shandong, China.

Variables	Total	Male	Female	χ^2^/t	*p*	df	Effect Size
Observations	4293 (100)	1800 (41.9)	2493 (58.1)				
General self-efficacy (GSE) score	27.2 ± 8.1	28.7 ± 7.7	26.1 ± 8.1	10.877	***0.000***	3986.044	0.335
Age	67.0 ± 4.0	67.2 ± 4.0	66.8 ± 4.0	3.712	***0.000***	4291	0.114
Marital				22.636	***0.000***	1	
Married	3691 (86.0)	1601 (88.9)	2090 (83.8)				
Others	602 (14.0)	199 (11.1)	403 (16.2)				
Education				583.678	***0.000***	3	
Illiteracy	1534 (35.7)	318 (17.7)	1216 (48.8)				
Primary school	1810 (42.2)	829 (46.1)	981 (39.4)				
Junior school	728 (17.0)	483 (26.8)	245 (9.8)				
Senior school or above	221 (5.1)	170 (9.4)	51 (2.0)				
Work				100.865	***0.000***	1	
Employed	1994(46.4)	998(55.4)	996(40.0)				
Unemployed	2299(53.6)	802(44.6)	1497(60.0)				
Income				222.199	***0.000***	3	
Q1	1145 (26.7)	334 (18.6)	811 (32.5)				
Q2	1029 (24.0)	373 (20.7)	656 (26.3)				
Q3	1047 (24.4)	459 (25.5)	588 (23.6)				
Q4	1072 (25.0)	634 (35.2)	438 (17.6)				
ADL				19.155	***0.000***	2	
I	3383 (78.8)	1475 (81.9)	1908 (76.5)				
II	759 (17.7)	266 (14.8)	493 (19.8)				
III	151 (3.5)	59 (3.3)	92 (3.7)				
Self-rated health				20.693	***0.000***	2	
Good	2261 (52.7)	1240 (49.7)	1021 (56.7)				
Normal	1170 (27.3)	716 (28.7)	454 (25.2)				
Bad	862 (20.1)	537 (21.5)	325 (18.1)				
Hypertension				33.083	***0.000***	1	
No	2413 (56.2)	1104 (61.3)	1309 (52.5)				
Yes	1880 (43.8)	696 (38.7)	1184 (47.5)				
Diabetes				43.618	***0.000***	1	
No	3719 (86.6)	1632 (90.7)	2087 (83.7)				
Yes	574 (13.4)	168 (9.3)	406 (16.3)				
Coronary				37.988	***0.000***	1	
No	3404 (79.3)	1508 (83.8)	1896 (76.1)				
Yes	889 (20.7)	292(16.2)	597(23.9)				
Psychological distress	15.7 ± 6.9	14.8 ± 6.7	16.3 ± 7.0	−6.674	***0.000***	3976.882	0.206
Personality				3.393	0.183	2	
Introvert	1299 (30.3)	572 (31.8)	727 (29.2)				
Extrovert	2152 (50.1)	882 (49.0)	1270 (50.9)				
Both	842 (19.6)	346 (19.2)	496 (19.9)				
Living arrangements				8.775	***0.003***	1	
Alone	444 (10.3)	157 (8.7)	287 (11.5)				
with children or others	3849 (89.7)	1643 (91.3)	2206 (88.5)				
Communication with children				23.236	***0.000***	1	
Yes	691 (16.1)	347 (19.3)	344 (13.8)				
No	3602 (83.9)	1453(80.7)	2149 (86.2)				
Intimate friends				4.639	***0.031***	1	
No	1171 (27.3)	522 (19.0)	649 (26.0)				
Yes	3122 (72.7)	1278 (71.0)	1844 (74.0)				
Satisfied with relationship				2.709	0.258	2	
Yes	4011(93.4)	1670 (92.8)	2341 (93.9)				
Moderate	237 (5.5)	107 (5.9)	130 (5.2)				
No	45(1.0)	23 (1.3)	22 (0.9)				

Bold and italicized numbers indicate *p* < 0.05.

**Table 2:** Determinants associated with GSE among the male young-old in rural Shandong, China.

Variables	Model 1				Model 2			
	β	SE	*p*	95%CI	β	SE	*p*	95%CI
Age								
	−0.230	0.045	***0.000***	(−0.319, −0.141)	−0.135	0.042	***0.001***	(−0.217, −0.054)
Marital (ref: others)								
married	−1.824	0.579	***0.002***	(−2.959, −0.689)	0.314	0.777	0.686	(−1.210,1.838)
Education (ref: Illiteracy)								
Primary school	2.252	0.499	***0.000***	(1.272,3.232)	1.756	0.443	***0.000***	(0.888,2.625)
Junior school	4.090	0.547	***0.000***	(3.018,5.163)	2.450	0.495	***0.000***	(1.479,3.421)
Senior school or above	4.794	0.719	***0.000***	(3.383,6.205)	2.282	0.660	***0.001***	(0.989,3.576)
Work (ref: employed)								
Unemployed	0.492	0.366	0.179	(−0.226,1.209)	NA	NA	NA	NA
Income (ref: Q1)								
Q2	0.651	0.569	0.253	(−0.465,1.768)	0.253	0.501	0.614	(−0.730,1.236)
Q3	0.928	0.543	0.088	(−0.138,1.994)	−0.436	0.482	0.366	(−1.383,0.510)
Q4	3.836	0.511	***0.000***	(2.834,4.838)	0.902	0.473	0.057	(−0.025,1.829)
ADL (ref: I)								
II	−3.979	0.492	***0.000***	(−4.944, −3.013)	−1.102	0.466	***0.018***	(−2.017, −0.188)
III	−10.397	0.981	***0.000***	(−12.321, −8.474)	−4.495	0.934	***0.000***	(−6.327, −2.663)
Self−rated health (ref: good)								
normal	−2.872	0.413	***0.000***	(−3.683, −2.061)	−1.246	0.392	***0.002***	(−2.015, −0.477)
bad	−6.366	0.467	***0.000***	(−7.281, −5.450)	−2.579	0.486	***0.000***	(−3.532, −1.626)
Hypertension (ref: no)								
yes	−1.926	0.371	***0.000***	(−2.653, −1.199)	−0.742	0.331	***0.025***	(−1.391, −0.092)
Diabetes (ref: no)								
yes	0.345	0.625	0.581	(−0.881,1.572)	NA	NA	NA	NA
Coronary (ref: no)								
yes	−1.635	0.492	***0.001***	(−2.600, −0.670)	0.751	0.444	0.091	(−0.119,1.621)
Psychological Distress								
	−0.435	0.025	***0.000***	(−0.484, −0.385)	−0.255	0.027	***0.000***	(−0.308, −0.202)
Personality (ref: Introvert)								
extrovert	3.807	0.404	***0.000***	(3.015,4.598)	1.754	0.369	***0.000***	(1.031,2.478)
both	0.996	0.512	0.052	(−0.009,2.000)	−0.030	0.455	0.948	(−0.921,0.862)
living arrangements (ref: alone)								
with children or other	1.727	0.643	***0.007***	(0.465,2.989)	0.136	0.866	0.875	(−1.563,1.836)
Frequent−communication with children (ref: no)								
yes	3.302	0.455	***0.000***	(2.410,4.193)	1.040	0.418	***0.013***	(0.220,1.860)
Intimate friends (ref: no)								
yes	4.693	0.385	***0.000***	(3.937,5.449)	2.020	0.374	***0.000***	(1.287,2.753)
Satisfied with relationship (ref: yes)								
moderate	−4.151	0.754	***0.000***	(−5.629, −2.673)	−1.450	0.675	***0.032***	(−2.773, −0.127)
no	−11.288	1.586	***0.000***	(−14.399, −8.176)	−3.201	1.451	***0.028***	(−6.047, −0.355)

Bold and italicized numbers indicate *p* < 0.05, R^2^ = 0.278, adjusted R^2^ = 0.269.

**Table 3:** Determinants associated with GSE among the female young-old in rural Shandong, China.

Variables	Model 1				Model 2			
	β	SE	*p*	95%CI	β	SE	*p*	95%CI
Age								
	−0.219	0.040	***0.000***	(−0.297, −0.141)	−0.119	0.038	***0.002***	(−0.194, −0.043)
Marital (ref: others)								
married	−1.273	0.441	***0.004***	(−2.138, −0.408)	−0.201	0.618	0.745	(−1.414,1.011)
Education (ref: Illiteracy)								
Primary school	1.750	0.345	***0.000***	(1.074,2.426)	1.002	0.321	***0.002***	(0.372,1.631)
Junior school	3.132	0.562	***0.000***	(2.029,4.235)	1.871	0.524	***0.000***	(0.843,2.898)
Senior school or above	5.010	1.148	***0.000***	(2.759,7.261)	3.416	1.055	***0.001***	(1.347,5.485)
Work (ref: employed)								
Unemployed	0.169	0.332	0.611	(−0.482,0.820)	NA	NA	NA	NA
Income (ref: Q1)								
Q2	0.074	0.423	0.862	(−0.756,0.904)	−0.194	0.384	0.614	(−0.947,0.559)
Q3	0.266	0.436	0.542	(−0.590,1.122)	−0.663	0.400	0.097	(−1.447,0.120)
Q4	2.777	0.478	***0.000***	(1.840,3.714)	1.001	0.447	***0.025***	(0.125,1.877)
ADL (ref: I)								
II	−3.396	0.399	***0.000***	(−4.179, −2.614)	−1.327	0.388	***0.001***	(−2.087, −0.567)
III	−7.812	0.843	***0.000***	(−9.465, −6.159)	−3.546	0.831	***0.000***	(−5.175, −1.918)
Self−rated health (ref: good)								
Normal	−3.314	0.369	***0.000***	(−4.038, −2.591)	−2.048	0.358	***0.000***	(−2.749, −1.347)
Bad	−4.791	0.406	***0.000***	(−5.587, −3.994)	−2.191	0.427	***0.000***	(−3.028, −1.354)
Hypertension (ref: no)								
Yes	−0.985	0.325	***0.002***	(−1.622, −0.347)	0.280	0.306	0.361	(−0.320,0.880)
Diabetes (ref: no)								
Yes	−0.504	0.440	0.253	(−1.367,0.360)	NA	NA	NA	NA
Coronary (ref: no)								
Yes	−1.732	0.380	***0.000***	(−2.476, −0.988)	−0.175	0.363	0.630	(−0.887,0.537)
Psychological Distress								
	−0.354	0.022	***0.000***	(−0.397, −0.310)	−0.210	0.024	***0.000***	(−0.256, −0.164)
Personality (ref: Introvert)								
Extrovert	3.791	0.370	***0.000***	(3.066,4.516)	1.989	0.354	***0.000***	(1.295,2.683)
Both	1.622	0.463	***0.000***	(0.714,2.530)	0.580	0.431	0.179	(−0.266,1.426)
Living arrangements (ref: alone)								
With children or other	1.496	0.509	***0.003***	(0.498,2.493)	0.750	0.715	0.294	(−0.652,2.152)
Frequent−communication with children (ref: no)								
Yes	2.950	0.468	***0.000***	(2.033,3.868)	0.766	0.437	0.080	(−0.090,1.622)
Intimate friends (ref: no)								
Yes	4.129	0.361	***0.000***	(3.420,4.837)	2.040	0.356	***0.000***	(1.342,2.738)
Satisfied with relationship (ref: yes)								
Moderate	−4.490	0.723	***0.000***	(−5.907, −3.073)	−1.935	0.676	***0.004***	(−3.262, −0.609)
No	−8.882	1.718	***0.000***	(−12.250, −5.513)	−2.294	1.632	0.160	(−5.493,0.906)

Bold and italicized numbers indicate *p* < 0.05, R^2^ = 0.200, adjusted R^2^ = 0.193.
